# A guide to selecting high-performing antibodies for Rab10 (UniProt ID: P61026) for use in western blot, immunoprecipitation, and immunofluorescence

**DOI:** 10.12688/f1000research.156209.1

**Published:** 2024-09-17

**Authors:** Vera Ruíz Moleón, Charles Alende, Maryam Fotouhi, Kathleen Southern, Riham Ayoubi, Carl Laflamme, NeuroSGC/YCharOS/EDDU collaborative group, ABIF consortium

**Affiliations:** 1 Department of Neurology and Neurosurgery, Structural Genomics Consortium, The Montreal Neurological Institute, McGill University, Montreal, Québec, H3A 2B4, Canada

**Keywords:** UniProt ID: P61026, RAB10, Rab10, antibody characterization, antibody validation, western blot, immunoprecipitation, immunofluorescence

## Abstract

Rab10 is a small GTPase involved in cargo transport from the trans-Golgi network to the plasma membrane and endocytic recycling back to the cell membrane. It has garnered significant interest in neurodegenerative disease research, particularly due to its phosphorylation by the LRRK2 kinase. This relationship underscores the importance of Rab10 in cellular processes related to disease pathology, specifically Parkinson’s disease. The accessibility of renewable and selective antibodies against Rab10 would advance research efforts, enabling further understanding of its function and implications in disease. Here, we have characterized eight Rab10 commercial antibodies for western blot, immunoprecipitation, and immunofluorescence using a standardized experimental protocol based on comparing read-outs in knockout cell lines and isogenic parental controls. These studies are part of a larger, collaborative initiative seeking to address antibody reproducibility issues by characterizing commercially available antibodies for human proteins and publishing the results openly as a resource for the scientific community. While use of antibodies and protocols vary between laboratories, we encourage readers to use this report as a guide to select the most appropriate antibodies for their specific needs.

## Introduction

The family of small GTPase Rabs are regulators of membrane trafficking, cycling between an active GTP-bound form and inactive GDP-bound form to recruit effector proteins and monitor vesicle formation and fusion with membranes. Rab10 is involved in the process of membrane trafficking from the trans-Golgi network to the plasma membrane. ^1^ Moreover, it regulates the transport of GLUT4 glucose-transport enriched vesicles to the plasma membrane. ^2^^–^^4^

Phosphorylation of Rab10 at Thr73 by Leucine-rich repeat kinase 2 (LRRK2) ^5^ regulates its activity. The characterization of phospho-Rab antibodies, including those targeting Rab10, has been conducted elsewhere. ^6^ LRRK2, a protein kinase associated with Parkinson’s disease, ^7^^,^^8^ has been shown to have increased kinase activity when subject to pathogenic mutations. ^9^ Elevated levels of phospho-Rab10 have been identified in neutrophils from Parkinson’s disease patients with LRRK2 mutations. ^10^ While inhibiting Rab10 is being explored as a potential therapeutic strategy, a deeper understanding of its mechanism is needed. This understanding could be greatly enhanced by the availability of selective and renewable Rab10 antibodies.

This research is part of a broader collaborative initiative in which academics, funders and commercial antibody manufacturers are working together to address antibody reproducibility issues by characterizing commercial antibodies for human proteins using standardized protocols, and openly sharing the data. ^11^^–^^13^ Here we evaluated the performance of eight commercial antibodies for Rab10 for use in western blot, immunoprecipitation, and immunofluorescence, enabling biochemical and cellular assessment of Rab10 properties and function. The platform for antibody characterization used to carry out this study was endorsed by a committee of industry academic representatives. It consists of identifying human cell lines with adequate target protein expression and the development/contribution of equivalent knockout (KO) cell lines, followed by antibody characterization procedures using commercially available antibodies against the corresponding protein. The standardized consensus antibody characterization protocols are openly available on Protocol Exchange, a preprint server (DOI: 10.21203/rs.3.pex-2607/v1). ^14^

The authors do not engage in result analysis or offer explicit antibody recommendations. Our primary aim is to deliver top-tier data to the scientific community, grounded in Open Science principles. This empowers experts to interpret the characterization data independently, enabling them to make informed choices regarding the most suitable antibodies for their specific experimental needs. Guidelines on how to interpret antibody characterization data found in this study are featured on the YCharOS gateway. ^15^

## Results and discussion

Our standard protocol involves comparing readouts from WT (wild type) and KO cells. ^16^^,^^17^ The first step was to identify a cell line(s) that expresses sufficient levels of a given protein to generate a measurable signal using antibodies. To this end, we examined the DepMap (Cancer Dependency Map Portal, RRID:SCR_017655) transcriptomics database to identify all cell lines that express the target at levels greater than 2.5 log _2_ (transcripts per million “TPM” + 1), which we have found to be a suitable cut-off. ^11^ The HAP1 expresses the Rab10 transcript at 7.9 log _2_ (TPM+1), which is above the average range of cancer cells analyzed. A *RAB10* KO HAP1 cell line was obtained from Horizon Discovery ( Table 1).

**Table 1.** Summary of the cell lines used.

Institution	Catalog number	RRID (Cellosaurus)	Cell line	Genotype
Horizon Discovery	C631	CVCL_Y019	HAP1	WT
Horizon Discovery	HZGHC008098c007	CVCL_D3U7	HAP1	*RAB10* KO

**Figure 1.**
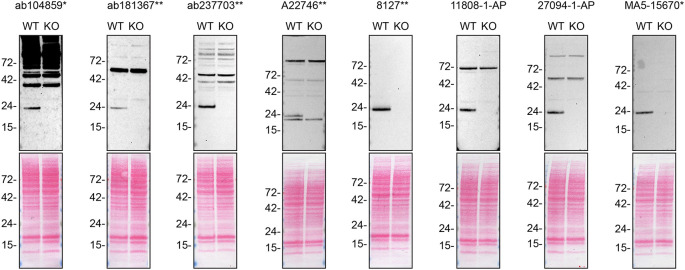
Rab10 antibody screening by western blot. Lysates of HAP1 WT and *RAB10* KO were prepared, and 40 μg of protein were processed for western blot with the indicated Rab10 antibodies. The Ponceau stained transfers of each blot are presented to show equal loading of WT and KO lysates and protein transfer efficiency from the acrylamide gels to the nitrocellulose membrane. Antibody dilutions were chosen according to the recommendations of the antibody supplier. All antibodies were used at 1/1000. Predicted band size: 22.5 kDa. *Monoclonal antibody, **Recombinant antibody.

For western blot experiments, WT and *RAB10* KO protein lysates were ran on SDS-PAGE, transferred onto nitrocellulose membranes, and then probed with eight Rab10 antibodies in parallel ( Table 2, Figure 1).

**Table 2.** Summary of the Rab10 antibodies tested.

Company	Catalog number	Lot number	RRID (Antibody registry)	Clonality	Clone ID	Host	Concentration (μg/μl)	Vendors recommended applications
Abcam	ab104859 *	1010852-4	AB_10711207	monoclonal	4E2	mouse	1.00	Wb, IF
Abcam	ab181367 **	GR3253726-3	AB_3107207	recombinant mono	EPR13242	rabbit	0.12	Wb
Abcam	ab237703 **	1014853-1	AB_2884879	recombinant mono	MJF-R23	rabbit	0.63	Wb, IP, IF
ABclonal	A22746 **	6100001129	AB_3086620	recombinant mono	ARC55653	rabbit	0.65	Wb, IF
Cell Signaling Technology	8127 **	3	AB_10828219	recombinant mono	D36C4	rabbit	0.25	Wb, IP, IF
Proteintech	11808-1-AP	00056330	AB_2173442	polyclonal	-	rabbit	0.27	Wb, IF
Proteintech	27094-1-AP	00050618	AB_2880752	polyclonal	-	rabbit	0.65	Wb, IF
Thermo Fisher Scientific	MA5-15670 *	ZA4191662	AB_10981668	monoclonal	4E2	mouse	n/a	Wb, IF

Wb = western blot; IF = immunofluorescence; IP = immunoprecipitation; n/a = not available.

* Monoclonal antibody.

** Recombinant antibody.

**Figure 2.**
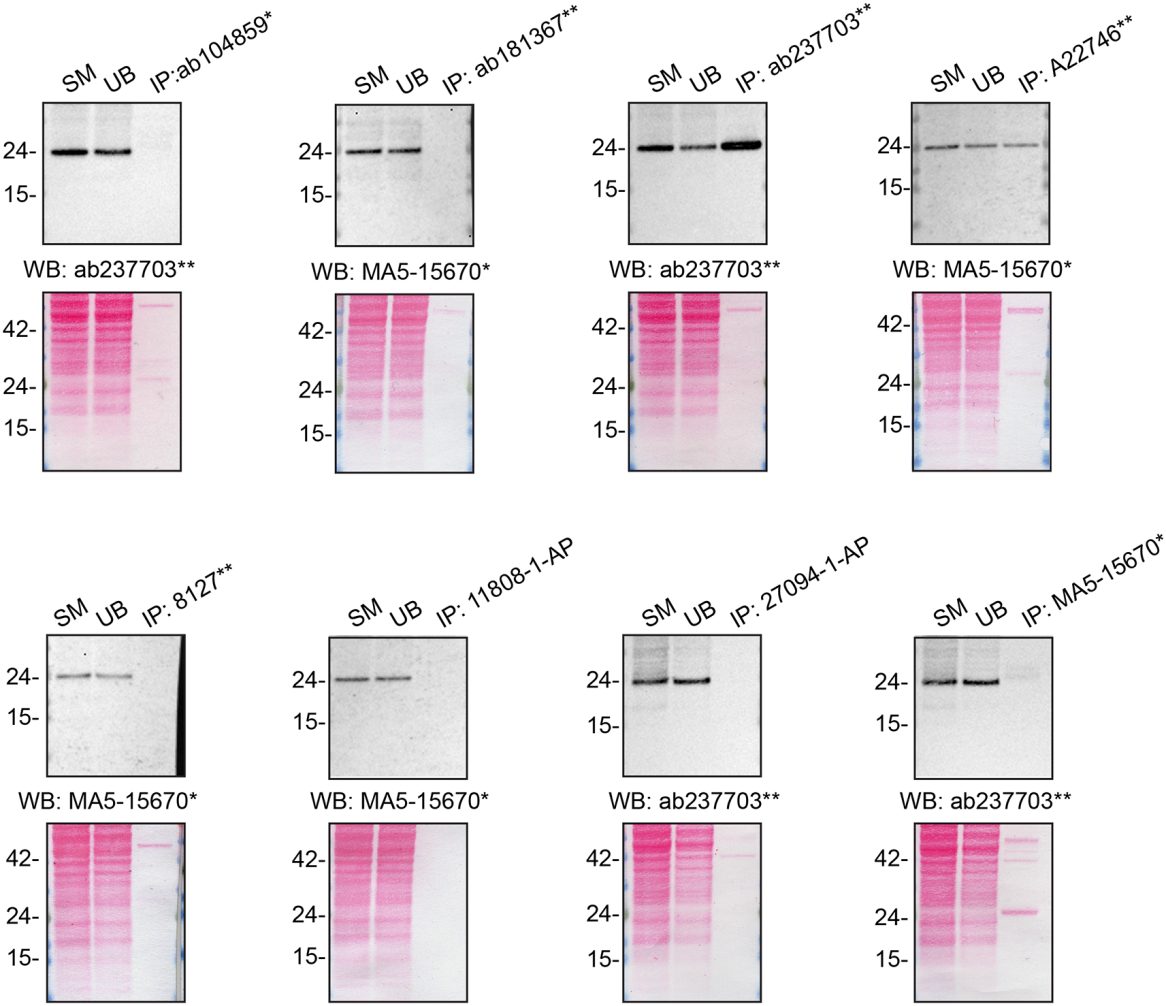
Rab10 antibody screening by immunoprecipitation. HAP1 lysates were prepared, and immunoprecipitation was performed using 1 mg of lysate and 2.0 μg of the indicated Rab10 antibodies pre-coupled to Dynabeads protein A or protein G. Samples were washed and processed for western blot with the indicated Rab10 antibody. For western blot, ab237703** and MA5-15670* were used at 1/500 and 1/1000, respectively. The Ponceau stained transfers of each blot are shown. SM = 4% starting material; UB = 4% unbound fraction; IP = immunoprecipitate. *Monoclonal antibody, **Recombinant antibody.

We then assessed the capability of all eight antibodies to capture Rab10 from HAP1 protein extracts using immunoprecipitation techniques, followed by western blot analysis. For the immunoblot step, a specific Rab10 antibody identified previously (refer to Figure 1) was selected. Equal amounts of the starting material (SM) and unbound fraction (UB), as well as the whole immunoprecipitate (IP) eluates were separated by SDS-PAGE ( Figure 2).

**Figure 3.**
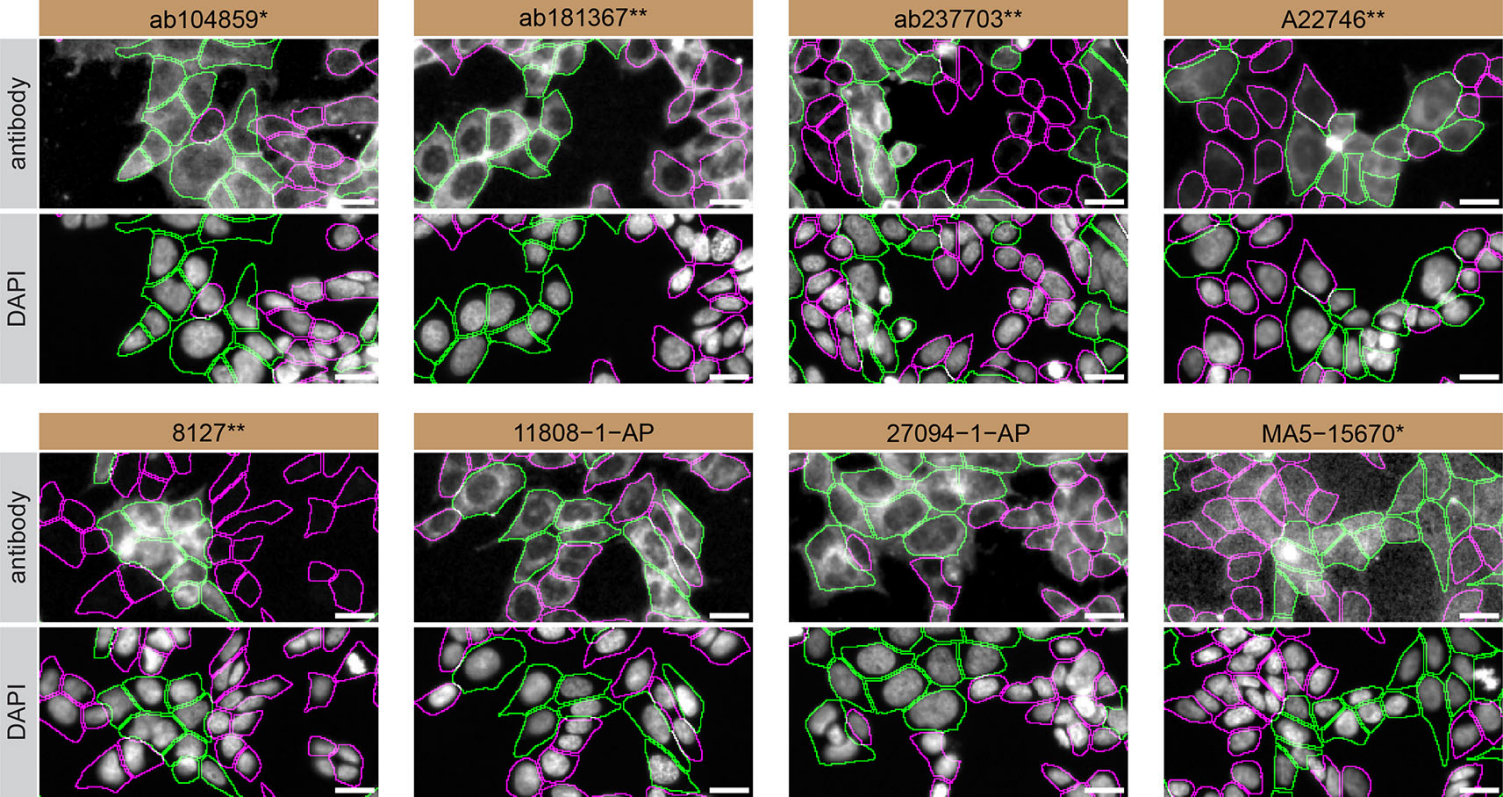
Rab10 antibody screening by immunofluorescence. HAP1 WT and *RAB10* KO cells were labelled with a green or a far-red fluorescent dye, respectively. WT and KO cells were mixed and plated to a 1:1 ratio on coverslips. Cells were stained with the indicated Rab10 antibodies and with the corresponding Alexa-fluor 555 coupled secondary antibody including DAPI. Acquisition of the blue (nucleus-DAPI), green (identification of WT cells), red (antibody staining) and far-red (identification of KO cells) channels was performed. Representative images of the merged blue and red (grayscale) channels are shown. WT and KO cells are outlined with green and magenta dashed line, respectively. When an antibody was recommended for immunofluorescence by the supplier, we tested it at the recommended dilution. The rest of the antibodies were tested at 1 and 2 μg/ml and the final concentration was selected based on the detection range of the microscope used and a quantitative analysis not shown here. Antibody dilution used: ab104859* at 1/200, ab181367** at 1/100, ab237703** at 1/500, A22746** at 1/600, 8127** at 1/50, 11808-1-AP at 1/400, 27094-1-AP at 1/600 and MA5-15670* at 1/500. Bars = 10 μm. *Monoclonal antibody, **Recombinant antibody.

For immunofluorescence, eight antibodies were screened using a mosaic strategy. First, HAP1 WT and *RAB10* KO cells were labelled with different fluorescent dyes in order to distinguish the two cell lines, and the Rab10 antibodies were evaluated. Both WT and KO lines imaged in the same field of view to reduce staining, imaging and image analysis bias ( Figure 3). Quantification of immunofluorescence intensity in hundreds of WT and KO cells was performed for each antibody tested, and the images presented in Figure 3 are representative of this analysis. ^14^

In conclusion, we have screened eight Rab10 commercial antibodies by western blot, immunoprecipitation, and immunofluorescence by comparing the signal produced by the antibodies in human HAP1 WT and *RAB10* KO cells. To assist viewers in interpreting antibody performance, Table 3 outlines various scenarios in which antibodies may perform in all three applications. Several high-quality and renewable antibodies that successfully detect Rab10 were identified in all applications. Researchers who wish to study Rab10 in a different species are encouraged to select high-quality antibodies, based on the results of this study, and investigate the predicted species reactivity of the manufacturer before extending their research.

The underlying data for this study can be found on Zenodo, an open-access repository for which YCharOS has its own collection of antibody characterization reports and corresponding dataset. ^18^^,^^19^

**Table 3.** Illustrations to assess antibody performance in western blot, immunoprecipitation and immunofluorescence.

Western blot	Immunoprecipitation	Immunofluorescence
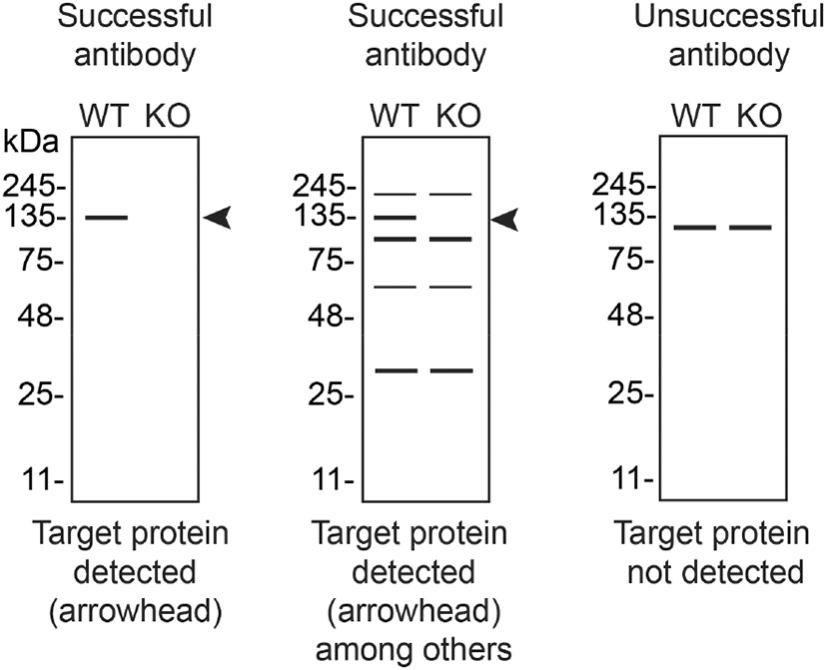	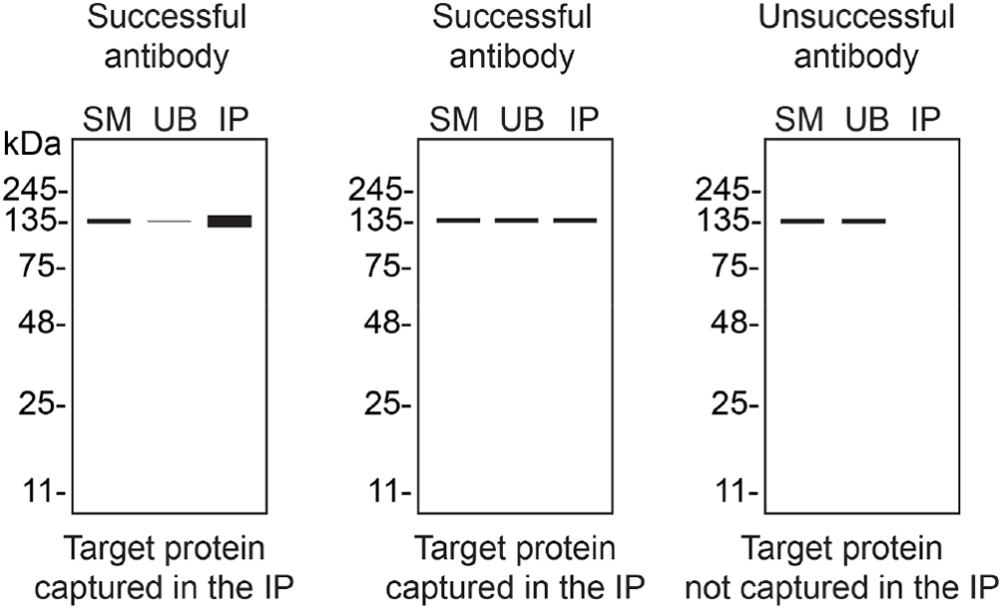	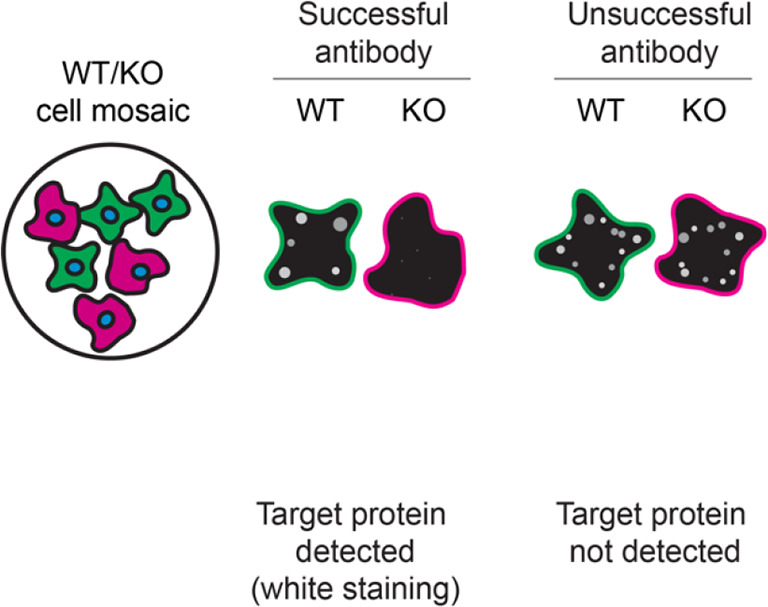

This table was reproduced with permission from Ayoubi et al., Elife, 2023. ^11^

### Limitations

Inherent limitations are associated with the antibody characterization platform used in this study. Firstly, the YCharOS project focuses on renewable (recombinant and monoclonal) antibodies and does not test all commercially available Rab10 antibodies. YCharOS partners make up approximately 80% of all renewable antibodies, but some top-cited polyclonal antibodies may not be available through these partners.

Secondly, the YCharOS effort employs a non-biased approach that is agnostic to the protein for which antibodies have been characterized. The aim is to provide objective data on antibody performance without preconceived notions about how antibodies should perform or the molecular weight that should be observed in western blot. As the authors are not experts on the Rab family of proteins or vesicle trafficking, only a brief overview of the protein’s function and its relevance in disease is provided. Rab10 experts are responsible for analyzing and interpreting observed banding patterns in western blots and subcellular localization in immunofluorescence.

Thirdly, YCharOS experiments are not performed in replicates primarily due to the use of multiple antibodies targeting various epitopes. Once a specific antibody is identified, it validates the protein expression of the intended target in the selected cell line, confirming the lack of protein expression in the KO cell line and supports conclusions regarding the specificity of the other antibodies. Moreover, some antibody clones are donated by 2-3 manufacturers (cross-licensed antibodies), effectively serving as replicates and enabling the validation of test reproducibility. All experiments are performed using master mixes, and meticulous attention is paid to sample preparation and experimental execution. In immunofluorescence, the use of two different concentrations serves to evaluate antibody specificity and can aid in assessing assay reliability. In instances where antibodies yield no signal, a repeat experiment is conducted following titration. Additionally, our independent data is performed subsequently to the antibody manufacturers internal validation process, therefore making our characterization process a repeat.

Lastly, as comprehensive and standardized procedures are respected, any conclusions remain confined to the experimental conditions and cell line used for this study. The use of a single cell type for evaluating antibody performance poses as a limitation, as factors such as target protein abundance significantly impact results. ^14^ Additionally, the use of cancer cell lines containing gene mutations poses a potential challenge, as these mutations may be within the epitope coding sequence or other regions of the gene responsible for the intended target. Such alterations can impact the binding affinity of antibodies. This represents an inherent limitation of any approach that employs cancer cell lines.

## Methods

The standardized protocols used to carry out this KO cell line-based antibody characterization platform was established and approved by a collaborative group of academics, industry researchers and antibody manufacturers. The detailed materials and step-by-step protocols used to characterize antibodies in western blot, immunoprecipitation and immunofluorescence are openly available on Protocol Exchange, a preprint server (DOI: 10.21203/rs.3.pex-2607/v1). ^14^ Brief descriptions of the experimental setup used to carry out this study can be found below.

### Cell lines and antibodies used

Cell lines used and primary antibodies tested in this study are listed in Tables 1 and 2, respectively. To ensure that the cell lines and antibodies are cited properly and can be easily identified, we have included their corresponding Research Resource Identifiers, or RRID. ^20^^,^^21^ HAP1 cells were cultured in DMEM high glucose (GE Healthcare cat. number SH30081.01) containing 10% fetal bovine serum (Wisent, cat. number 080450), 2 mM L-glutamine (Wisent cat. number 609-065, 100 IU penicillin and 100 μg/ml streptomycin (Wisent cat. number 450201).

Peroxidase-conjugated goat anti-rabbit and anti-mouse antibodies are from Thermo Fisher Scientific (cat. number 65-6120 and 62-6520). Alexa-555-conjugated goat anti-rabbit and anti-mouse secondary antibodies are from Thermo Fisher Scientific (cat. number A-21429 and A-21424). Peroxidase-conjugated Protein A for IP detection is from MilliporeSigma, cat. number P8651. Peroxidase-conjugated anti-mouse IgG for IP detection is from Abcam, cat. number ab131368.

### Antibody screening by western blot

HAP1 WT and *RAB10* KO cells were collected in RIPA buffer (25 mM Tris-HCl pH 7.6, 150 mM NaCl, 1% NP-40, 1% sodium deoxycholate, 0.1% SDS) (Thermo Fisher Scientific, cat. number 89901) supplemented with 1× protease inhibitor cocktail mix (MilliporeSigma, cat. number P8340). Lysates were sonicated briefly and incubated 30 min on ice. Lysates were spun at ~110,000 × *g* for 15 min at 4°C and equal protein aliquots of the supernatants were analyzed by SDS-PAGE and western blot. BLUelf prestained protein ladder (GeneDireX, cat. number PM008-0500) was used.

Western blots were performed with precast midi 10% Bis-Tris polyacrylamide gels from Thermo Fisher Scientific (cat. number WG1201BOX) ran with MES SDS buffer (Thermo Fisher Scientific, cat. number NP000202), loaded in LDS sample buffer (Thermo Fisher Scientific, cat. number NP0008) with 1× sample reducing agent (Thermo Fisher Scientific, cat. number NP0009) transferred on nitrocellulose membranes. Proteins on the blots were visualized with Ponceau S staining (Thermo Fisher Scientific, cat. number BP103-10) which is scanned to show together with individual western blot. Blots were blocked with 5% milk for 1 hr, and antibodies were incubated overnight at 4°C with 5% milk in TBS with 0,1% Tween 20 (TBST) (Cell Signalling Technology, cat. number 9997). Following three washes with TBST, the peroxidase conjugated secondary antibody was incubated at a dilution of ~0.2 μg/ml in TBST with 5% milk for 1 hr at room temperature followed by three washes with TBST. Membranes were incubated with Pierce ECL (Thermo Fisher Scientific, cat. number 32106) or Clarity Western ECL Substrate (Bio-Rad, cat. number 1705061) prior to detection with the iBright™ CL1500 Imaging System (Thermo Fisher Scientific (cat. number A44240).

### Antibody screening by immunoprecipitation

Antibody-bead conjugates were prepared by adding 2 μg to 500 μl of Pierce IP Lysis Buffer (Thermo Fisher Scientific, cat. number 87788) in a microcentrifuge tube, together with 30 μl of Dynabeads protein A - (for rabbit antibodies) or protein G - (for mouse antibodies) (Thermo Fisher Scientific, cat. number 10002D and 10004D, respectively). Tubes were rocked for ~1 hr at 4°C followed by two washes to remove unbound antibodies.

HAP1 WT cells were collected Pierce IP buffer (25 mM Tris-HCl pH 7.4, 150 mM NaCl, 1 mM EDTA, 1% NP-40 and 5% glycerol) supplemented with protease inhibitor. Lysates were rocked for 30 min at 4°C and spun at 110,000 × *g* for 15 min at 4°C. 0.5 ml aliquots at 2.0 mg/ml of lysate were incubated with an antibody-bead conjugate for ~1 hr at 4°C. The unbound fractions were collected, and beads were subsequently washed three times with 1.0 ml of IP lysis buffer and processed for SDS-PAGE and western blot on a precast midi 10% Bis-Tris polyacrylamide gels. Protein A: HRP was used as a secondary antibody at a dilution of 2 μg/ml when the rabbit antibody ab237703** was used for western blot to detect the IP. Anti-mouse IgG for IP: HRP was used as a secondary antibody at a concentration of 0.3 μg/ml when the mouse antibody MA5-15670* was used for western blot to detect the IP.

### Antibody screening by immunofluorescence

HAP1 WT and *RAB10* KO cells were labelled with a green and a far-red fluorescence dye, respectively (Thermo Fisher Scientific, cat. number C2925 and C34565). The nuclei were labelled with DAPI (Thermo Fisher Scientific, cat. Number D3571) fluorescent stain. WT and KO cells were plated on 96-well plate with optically clear flat-bottom (Perkin Elmer, cat. number 6055300) as a mosaic and incubated for 24 hrs in a cell culture incubator at 37 ^o^C, 5% CO _2_. Cells were fixed in 4% paraformaldehyde (PFA) (VWR, cat. number 100503-917) in phosphate buffered saline (PBS) (Wisent, cat. number 311-010-CL). Cells were permeabilized in PBS with 0.1% Triton X-100 (Thermo Fisher Scientific, cat. number BP151-500) for 10 min at room temperature and blocked with PBS with 5% BSA, 5% goat serum (Gibco, cat. number 16210-064) and 0.01% Triton X-100 for 30 min at room temperature. Cells were incubated with IF buffer (PBS, 5% BSA, 0.01% Triton X-100) containing the primary Rab10 antibodies overnight at 4°C. Cells were then washed 3 × 10 min with IF buffer and incubated with corresponding Alexa Fluor 555-conjugated secondary antibodies in IF buffer at a dilution of 1.0 μg/ml for 1 hr at room temperature with DAPI. Cells were washed 3 × 10 min with IF buffer and once with PBS.

Images were acquired on an ImageXpress micro confocal high-content microscopy system (Molecular Devices), using a 20x NA 0.95 water immersion objective and scientific CMOS cameras, equipped with 395, 475, 555 and 635 nm solid state LED lights (lumencor Aura III light engine) and bandpass filters to excite DAPI, Cellmask Green, Alexa-555 and Cellmask Red, respectively. Images had pixel sizes of 0.68 x 0.68 microns, and a z-interval of 4 microns. For analysis and visualization, shading correction (shade only) was carried out for all images. Then, maximum intensity projections were generated using 3 z-slices. Segmentation was carried out separately on maximum intensity projections of Cellmask channels using CellPose 1.0, and masks were used to generate outlines and for intensity quantification. ^22^ Figures were assembled with Adobe Illustrator.

## Data Availability

Zenodo: Antibody Characterization Report Rab10, https://doi.org/10.5281/zenodo.13684961. ^18^ Zenodo: Dataset for the Rab10 antibody screening study, https://doi.org/10.5281/zenodo.13685286. ^19^ Data are available under the terms of the Creative Commons Attribution 4.0 International license (CC-BY 4.0).
