# Supplementary material for: A guide to selecting high-performing antibodies for Rab10 (UniProt ID: P61026) for use in western blot, immunoprecipitation, and immunofluorescence
Source: F1000Res. 2024 Sep 17;13:1061. [Version 1] doi: 10.12688/f1000research.156209.1 (PMC12174638; doi:10.12688/f1000research.156209.1)
Supplement: Peer review history

# Reviewer report: Reviewer response for version 1

**DOI:** 10.5256/f1000research.171489.r325290

**Published:** 2024-10-10

**Authors:** Sian E. Evans

**Affiliations:** 1 University of Leicester, Leicester, England, UK

The data note provides a detailed description of the characterisation of eight commercial antibodies targeting Rab10. The study evaluates their suitability for western blot, immunoprecipitation, and immunofluorescence using a standardised experimental protocol.

 The aim is to assess antibody performance for Rab10 using a wild-type and knock-out cell line. The materials and methods are well-documented, and a link to the standardised antibody characterisation protocols is provided for further reference (DOI: 10.21203/rs.3.pex-2607/v1).

 The results and discussion section is concise and coherent. The authors justify their choice of using the HAP1 cell line and reference the database they utilized, which will be beneficial for other researchers. All images and tables are presented clearly. The inclusion of Table 3, which demonstrates the distinction between successful and unsuccessful antibody performance, is particularly helpful for researchers in interpreting the data.

Are sufficient details of methods and materials provided to allow replication by others?

Yes

Is the rationale for creating the dataset(s) clearly described?

Yes

Are the datasets clearly presented in a useable and accessible format?

Yes

Are the protocols appropriate and is the work technically sound?

Yes

Reviewer Expertise:

Oncology, Western Blotting, ELISA, Immuno-fluorescence, Cell-based assays.

I confirm that I have read this submission and believe that I have an appropriate level of expertise to confirm that it is of an acceptable scientific standard.

# Reviewer report: Reviewer response for version 1

**DOI:** 10.5256/f1000research.171489.r325289

**Published:** 2024-10-10

**Authors:** Jan Voskuil

**Affiliations:** 1 Aeonian Biotech Netherlands, Leiden, The Netherlands

Table 3 was not visible, broken images.

 The authors delivered an excellent approach to validate commercial antibodies by comparing their generated signals from a suitable cell line, proven to express the target gene sufficiently for antibody detection, with its cognate knock-out derivative cell line.

 In their approach, they analysed the antibody performance in Western blot (WB), in immuno-precipitation (IP), and in immunocytochemistry (staining of entire cells detected by immunofluorescence, IF).

 Although the objective of the authors are to present their approach as a method of finding high performing antibodies from the market, the data themselves are left without scientific assessment. I think this paper will benefit from an extra paragraph to discuss the quality of the individual data. For example, some antibodies show extra bands in WB, and it is not clear whether these bands would disappear upon further antibody dilution (without loss of specific signal), or that all the bands, the correct and the wrong ones, would equally fade by further antibody dilution. The target Rab10 is located in vesicular membranes and perinuclear. Its location in the IF data is not very clear in many cases. A few sentences on the quality of the data would be desirable and put some extra scientific weight to the article.

 The authors suggest that several antibodies worked well across all three applications, but I could only find one of them. Table 3 was inaccessible to me.

 This paper is extremely useful for scientists needing a good Rab10 antibody for either WB, IP or ICC. I highly commend this paper for indexing, and I hope my remarks will be addressed in the final version.

Are sufficient details of methods and materials provided to allow replication by others?

Yes

Is the rationale for creating the dataset(s) clearly described?

Yes

Are the datasets clearly presented in a useable and accessible format?

Partly

Are the protocols appropriate and is the work technically sound?

Yes

Reviewer Expertise:

Life-time career expertise on antibody characterisation and validation in all immunoassays. Both experience as an academic scientist, and as a commercial provider.

I confirm that I have read this submission and believe that I have an appropriate level of expertise to confirm that it is of an acceptable scientific standard.
